# Supplementary material for: Genetic signatures of population bottlenecks, relatedness, and inbreeding highlight recent and novel conservation concerns in the Egyptian vulture
Source: PeerJ. 2021 Mar 25;9:e11139. doi: 10.7717/peerj.11139 (PMC8005290; doi:10.7717/peerj.11139)
Supplement: Supplemental Information 1 [file peerj-09-11139-s001.docx]

**Supplementary materials**

**Genetic signatures of population bottlenecks, relatedness, and inbreeding alert on recent and novel conservation concerns in the Egyptian vulture**

Guillermo Blanco and Francisco Morinha

**Table S1** Summary of microsatellite data per locus for the three datasets analysed (all individuals; offspring 2006; and offspring 2018). For offspring 2006 and 2018 only one individual per nest was considered (listed in Table S2 and S3).

|  |  | **All individuals (n=42)** | | | | | |  | **Offspring 2006 (n=15)** | | | | | |  | **Offspring 2018 (n=10)** | | | | | |
| --- | --- | --- | --- | --- | --- | --- | --- | --- | --- | --- | --- | --- | --- | --- | --- | --- | --- | --- | --- | --- | --- |
| **Locus** | **Allele size** | ***N_A_*** | ***A_R_*** | ***H_O_*** | ***H_E_*** | ***P*** | ***F_IS_*** |  | ***N_A_*** | ***A_R_*** | ***H_O_*** | ***H_E_*** | ***P*** | ***F_IS_*** |  | ***N_A_*** | ***A_R_*** | ***H_O_*** | ***H_E_*** | ***P*** | ***F_IS_*** |
| BV9 | 224-232 | 5 | 4.38 | 0.69 | 0.68 | 0.12 | 0.00 |  | 5 | 4.89 | 1.00 | 0.75 | 0.20 | -0.30 |  | 3 | 3.00 | 0.50 | 0.51 | 1.00 | 0.06 |
| BV13 | 171-181 | 5 | 3.65 | 0.76 | 0.65 | 0.43 | -0.16 |  | 4 | 3.90 | 0.80 | 0.70 | 0.67 | -0.12 |  | 4 | 4.00 | 0.80 | 0.67 | 0.88 | -0.14 |
| BV14 | 163-165 | 2 | 2.00 | 0.45 | 0.44 | 1.00 | -0.03 |  | 2 | 2.00 | 0.27 | 0.39 | 0.23 | 0.35 |  | 2 | 2.00 | 0.70 | 0.46 | 0.22 | -0.50 |
| BV20 | 135-137 | 2 | 2.00 | 0.60 | 0.50 | 0.35 | -0.19 |  | 2 | 2.00 | 0.53 | 0.48 | 1.00 | -0.08 |  | 2 | 2.00 | 0.70 | 0.50 | 0.52 | -0.37 |
| NP39 | 278-298 | 8 | 6.70 | 0.76 | 0.82 | 0.01 | 0.09 |  | 7 | 6.32 | 0.67 | 0.82 | 0.07 | 0.22 |  | 8 | 8.00 | 0.80 | 0.84 | 0.06 | 0.09 |
| NP51 | 143-155 | 4 | 1.90 | 0.10 | 0.09 | 1.00 | -0.02 |  | 4 | 3.23 | 0.27 | 0.24 | 1.00 | -0.07 |  | 1 | 1.00 | 0.00 | 0.00 | - | - |
| NP93 | 244-250 | 3 | 2.99 | 0.67 | 0.62 | 0.97 | -0.06 |  | 3 | 3.00 | 0.60 | 0.66 | 0.84 | 0.12 |  | 3 | 3.00 | 0.80 | 0.57 | 0.44 | -0.37 |
| NP140 | 396-400 | 3 | 2.67 | 0.31 | 0.54 | <0.01 | 0.44 |  | 3 | 2.67 | 0.53 | 0.41 | 0.63 | -0.28 |  | 3 | 3.00 | 0.20 | 0.58 | **<0.01** | 0.68 |
| NP141 | 286-302 | 6 | 4.67 | 0.55 | 0.67 | <0.01 | 0.19 |  | 6 | 5.27 | 0.67 | 0.71 | 0.76 | 0.09 |  | 4 | 4.00 | 0.50 | 0.65 | 0.04 | 0.28 |
| NP155 | 349-355 | 4 | 3.62 | 0.52 | 0.54 | 0.06 | 0.03 |  | 4 | 3.89 | 0.67 | 0.62 | <0.01 | -0.04 |  | 3 | 3.00 | 0.60 | 0.45 | 1.00 | -0.30 |
| NP163 | 230-240 | 5 | 4.35 | 0.69 | 0.73 | 0.16 | 0.07 |  | 4 | 3.96 | 0.87 | 0.65 | 0.17 | -0.30 |  | 4 | 4.00 | 0.70 | 0.72 | 0.34 | 0.08 |
| NP166 | 190-198 | 5 | 4.55 | 0.64 | 0.64 | 0.21 | 0.01 |  | 5 | 4.78 | 0.67 | 0.60 | 0.96 | -0.09 |  | 4 | 4.00 | 0.50 | 0.67 | 0.08 | 0.30 |
| NP229 | 167-187 | 6 | 5.13 | 0.79 | 0.75 | 0.63 | -0.03 |  | 4 | 3.67 | 0.67 | 0.68 | 1.00 | 0.06 |  | 6 | 6.00 | 0.80 | 0.77 | 0.36 | 0.01 |
| NP249 | 241-249 | 4 | 3.85 | 0.64 | 0.65 | 0.30 | 0.02 |  | 4 | 3.91 | 0.53 | 0.48 | 0.82 | -0.08 |  | 4 | 4.00 | 0.70 | 0.64 | 0.57 | -0.05 |
| NP257 | 108-110 | 2 | 1.82 | 0.14 | 0.13 | 1.00 | -0.06 |  | 2 | 1.99 | 0.27 | 0.23 | 1.00 | -0.12 |  | 2 | 2.00 | 0.10 | 0.10 | - | - |
| NP259 | 121-127 | 4 | 3.23 | 0.60 | 0.64 | 0.01 | 0.08 |  | 3 | 3.00 | 0.53 | 0.62 | 0.14 | 0.18 |  | 4 | 4.00 | 0.60 | 0.67 | 0.09 | 0.16 |
| NP296 | 172-188 | 7 | 6.05 | 0.86 | 0.78 | 0.07 | -0.09 |  | 7 | 6.35 | 1.00 | 0.76 | 0.36 | -0.29 |  | 6 | 6.00 | 0.70 | 0.81 | 0.16 | 0.18 |
| **Mean** | - | 4.41 | 3.74 | 0.57 | 0.58 | - | 0.02 |  | 4.06 | 3.81 | 0.62 | 0.58 | - | -0.04 |  | 3.71 | 3.71 | 0.57 | 0.56 | - | 0.01 |
| *N_A_* - number of alleles, *H_O_* - observed heterozygosity, *H_E_* - expected heterozygosity (GenAlEx v6.5); *A_R_* - Allelic richness (FSTAT v2.9.4); *P* - exact probability for expected Hardy Weinberg equilibrium conditions for each locus/population combination, *F_IS_* - Weir & Cockerham (1984) (GENEPOP V4.2.2). **Values in bold:** deviations from HWE significant after Bonferroni correction. | | | | | | | | | | | | | | | | | | | | | |

**Table S2** Results of Fisher’s exact probability tests of differences in allele frequencies between offspring 2006 and 2018.

| **Locus** | ***P*-value** |
| --- | --- |
| BV9 | 0.07 |
| BV13 | 0.50 |
| BV14 | 0.75 |
| BV20 | 0.78 |
| NP39 | 0.70 |
| NP51 | 0.51 |
| NP93 | 0.20 |
| NP140 | 0.37 |
| NP141 | 0.39 |
| NP155 | 0.09 |
| NP163 | 0.44 |
| NP166 | 0.47 |
| NP229 | 0.19 |
| NP249 | 0.32 |
| NP257 | 0.40 |
| NP259 | 0.42 |
| NP296 | 0.65 |
| Overall | 0.44 |

**Table S3** Genetic relatedness estimated for the group of know full-siblings and the group of all Egyptian vultures included in this study. *n* represents the number of pairwise comparisons.

| **Groups** | **Relatedness values (mean values ± standard deviation)** | | | | |
| --- | --- | --- | --- | --- | --- |
|  | Wang | Lynch & Li | Lynch & Ritland | Ritland | Queller & Goodnight |
| Full-sibs (*n*=19) | 0.59±0.13 | 0.60±0.12 | 0.43±0.17 | 0.28±0.12 | 0.60±0.12 |
| All samples (*n*=861) | -0.01±0.26 | -0.01±0.26 | -0.02±0.20 | -0.02±0.18 | -0.02±0.24 |

**Table S4** COANCESTRY pairwise correlations obtained for the five genetic relatedness estimators tested using the values estimated for the group of all Egyptian vultures.

|  | **Wang** | **Lynch & Li** | **Lynch & Ritland** | **Ritland** | **Queller & Goodnight** |
| --- | --- | --- | --- | --- | --- |
| **Wang** | 1.00 |  |  |  |  |
| **Lynch & Li** | 0.97 | 1.00 |  |  |  |
| **Lynch & Ritland** | 0.85 | 0.87 | 1.00 |  |  |
| **Ritland** | 0.75 | 0.80 | 0.88 | 1.00 |  |
| **Queller& Goodnight** | 0.89 | 0.94 | 0.92 | 0.87 | 1.00 |

**Table S5** Levels of homozygosity by loci (*HL*) in juvenile Egyptian vultures (offspring 2006, 2018 and 2020) calculating considering only one individual per nest.

| **Ind.** | **Year** | **HL** |
| --- | --- | --- |
| 24N | 2006 | 0.35 |
| 24P | 2006 | 0.40 |
| 24R | 2006 | 0.42 |
| 24T | 2006 | 0.30 |
| 24V | 2006 | 0.32 |
| 24X | 2006 | 0.45 |
| 24W | 2006 | 0.52 |
| 9037000 | 2006 | 0.21 |
| 24A | 2006 | 0.19 |
| 24F | 2006 | 0.27 |
| 24H | 2006 | 0.38 |
| 24J | 2006 | 0.28 |
| 24M | 2006 | 0.33 |
| G15746 | 2006 | 0.26 |
| G15749 | 2006 | 0.22 |
| 9MC | 2018 | 0.48 |
| 9MA | 2018 | 0.42 |
| 9LJ | 2018 | 0.59 |
| 9LL | 2018 | 0.48 |
| 9LM | 2018 | 0.29 |
| 9LN | 2018 | 0.35 |
| 9LP | 2018 | 0.26 |
| 9LR | 2018 | 0.31 |
| 9LU | 2018 | 0.33 |
| 9LW | 2018 | 0.36 |
| 9MF | 2020 | 0.54 |

**Table S6** Results of the M-ratio analysis. For the Mode-shift test, modes obtained for each population are indicated. Values of the observed M-ratio (M) and critical ratio (Mc) were estimated for three values of pre-bottleneck θ that correspond to Ne values of 50, 500, 2500, and 5000, respectively. Values in bold suggest demographic bottleneck events.

|  | *M-ratio* (M_c_) | | | | | |
| --- | --- | --- | --- | --- | --- | --- |
| Year | M | θ = 0.1 | θ = 1 | θ = 5 | | θ = 10 |
| 2006 | 0.901 | 0.801 | 0.738 | 0.647 | | 0.610 |
| 2018 | 0.921 | 0.799 | 0.727 | 0.617 | | 0.568 |
| 2006+2018* | 0.906 | 0.802 | 0.733 | 0.666 | | 0.641 |
| *Only individuals from different territories | | | | |  |  |


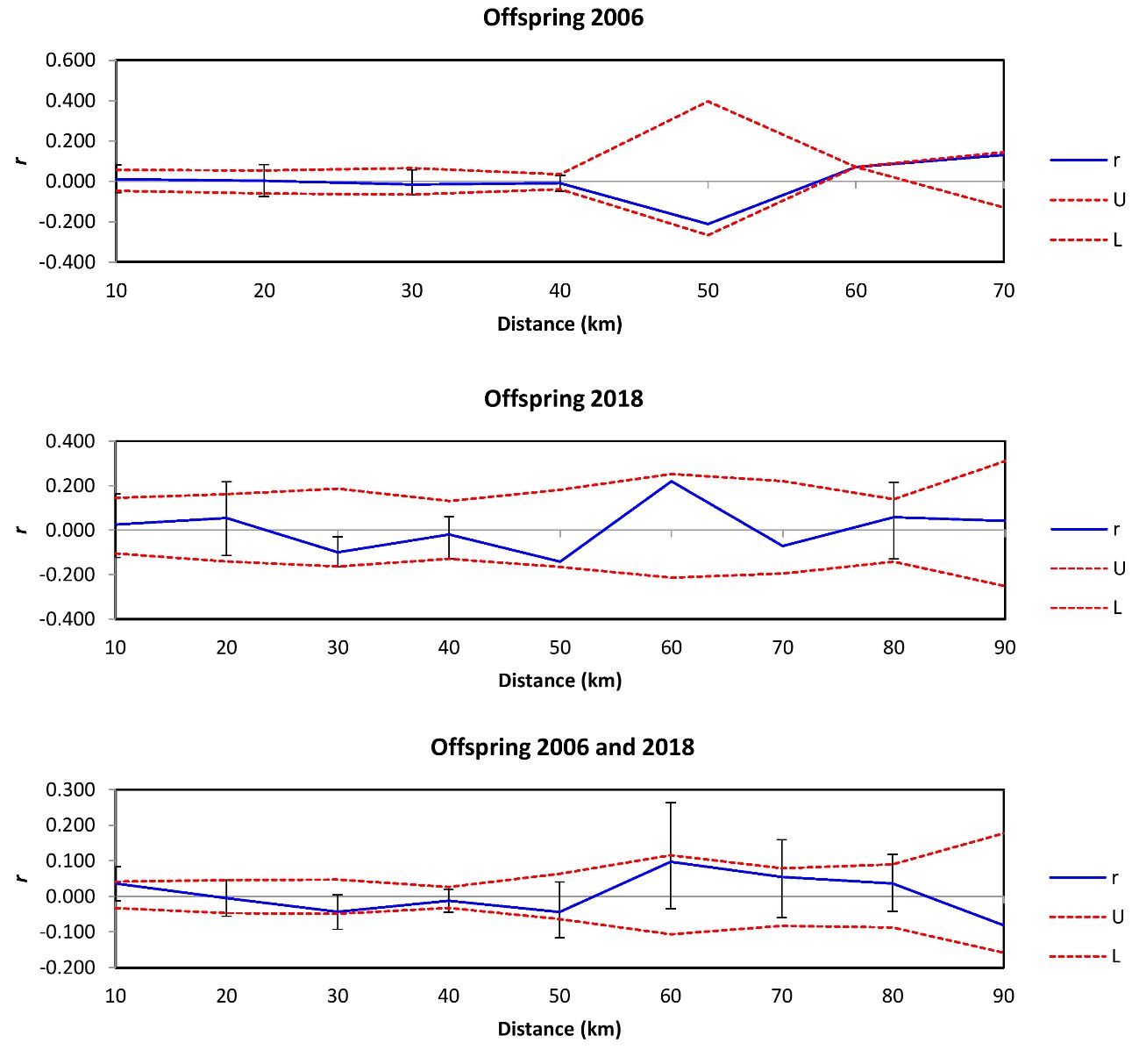


**Figure S1** Spatial autocorrelation analyses of the genetic correlation coefficient (*r*) as a function of distance for Egyptian vulture’s offspring from 2006 and 2018. Upper (U) and lower (L) dashed lines represent 95% confidence limits for a null hypothesis of an random spatial distribution of genotypes. Error bars indicate 95% confidence intervals around each *r*.

**
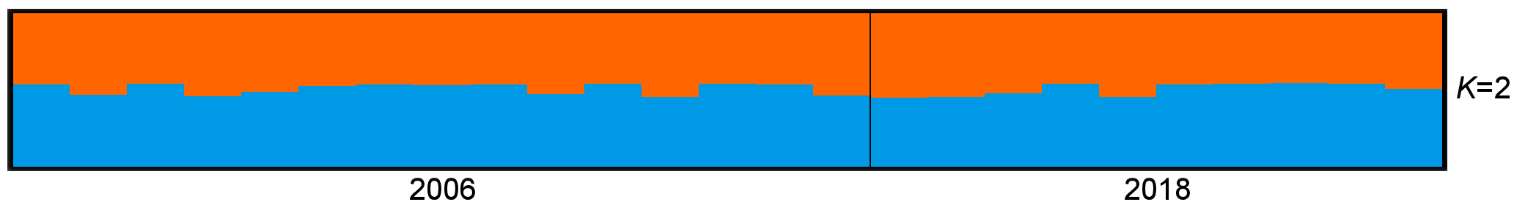
Figure S2** Genetic structure inferred with the program STRUCTURE v.2.3.4 by using Egyptian vulture microsatellite data (offspring 2006 and 2018). Each vertical line corresponds to one individual and colours represent the proportional posterior probability of assignment to each cluster.
